# Cone-Beam Computed Tomography Assessment of Root Canal Morphological Variations in Maxillary Second Premolars

**DOI:** 10.7759/cureus.88731

**Published:** 2025-07-25

**Authors:** Anam Masood, Sadiq Amin Ahmed Rana, Mobeen Akhtar, Syed Midhat Batool, Afshan Manzoor, Muhammad Ali

**Affiliations:** 1 Operative Dentistry, Bakhtawar Amin Medical and Dental College, Multan, PAK; 2 Operative Dentistry, Sadiq Amin Medical and Dental Complex, Multan, PAK; 3 Operative Dentistry, Nishtar Institute of Dentistry, Multan, PAK; 4 Periodontology, Bakhtawar Amin Medical and Dental College, Multan, PAK; 5 Community Dentistry, Bakhtawar Amin Medical and Dental College, Multan, PAK

**Keywords:** cbct, root canal morphology, root canal treatment, second premolar, vertucci’s classification system

## Abstract

Background and objective

A comprehensive understanding of root canal morphology and its anatomical variations is critical for the success of root canal treatment (RCT). Unrecognized variations may lead to incomplete cleaning, shaping, and obturation, potentially resulting in treatment failure. This study aimed to evaluate the frequency and distribution of root canal configuration types in maxillary second premolars (Mx2P) and their association with gender using cone-beam computed tomography (CBCT).

Materials and methods

This retrospective cross-sectional study analyzed 384 CBCT scans of fully developed, untreated Mx2Ps in patients aged 18 to 65 years. Teeth with restorations, prior endodontic treatment, gross caries, fractures, ankylosis, or pulpal calcifications were excluded. Root canal morphology was classified according to Vertucci’s classification system using the Carestream Dental Imaging Software 3D Module v2.4. Gender-based comparisons were conducted using the chi-square test.

Results

Scans from 219 patients (114 males (52.1%), 105 females (47.9%)) with a mean age of 42.35 ± 13.79 years were included. The most prevalent canal configuration was Vertucci’s Type I (n = 240, 62.5%), followed by Type II (n = 96, 25.0%), Type IV (n = 30, 7.8%), Type VI (n = 3, 0.8%), Type VII (n =3, 0.8%), Type III (n = 7, 1.8%), Type V (n = 4, 1.0%), and Type VIII (n = 1, 0.3%). No statistically significant association was found between root canal morphology and gender (p = 0.915).

Conclusion

Vertucci’s Type I was the most common root canal configuration in maxillary second premolars. The findings highlight the importance of thorough radiographic assessment using CBCT to identify anatomical variations for effective endodontic treatment planning.

## Introduction

The dental pulp canal exhibits a wide range of anatomical variations in shape and configuration across dentition. For both surgical and non-surgical endodontic procedures, the success of treatment hinges on a thorough understanding of dental morphology, accurate interpretation of radiographs, appropriate access cavity preparation, and precise location of root canal orifices. Optimal outcomes are achieved when the root canal system is adequately accessed, meticulously cleaned and shaped, effectively disinfected, and hermetically obturated [1].

Inadequate access cavity preparation can hinder canal location and instrumentation, compromising the overall quality of treatment. The root canal system is inherently complex and often unpredictable, necessitating detailed knowledge of tooth anatomy and careful radiographic evaluation. While periapical radiographs provide initial insights, they often fail to reveal the true three-dimensional architecture of the root canal system. More comprehensive imaging modalities, such as multiple angulated radiographs or cone-beam computed tomography (CBCT), offer superior visualization of root canal configurations [2].

Root canal morphology varies significantly not only across different populations but also among individuals within the same population. Clinicians must be well-versed in these anatomical variations and potential anomalies to avoid missed canals, which can lead to persistent periapical infections due to retained microorganisms and necrotic tissue. In fact, insufficient understanding of root canal anatomy is a leading cause of endodontic treatment failure [3].

Accurate clinical and radiographic assessments are essential for determining the number, configuration, and spatial orientation of root canals. Maxillary second premolars (Mx2P) are among the most frequently treated teeth in endodontic practice and are known for their morphological variability [4]. The canal system of the Mx2P may exhibit multiple branches, with bifurcations, trifurcations, and rejoining patterns before terminating at the apical foramen. These teeth may present with one to three canals and one to three roots, with a buccolingually broader root profile than mesiodistally. In cases with two canals, a wider buccolingual access cavity is necessary to ensure straight-line access. Western populations predominantly exhibit 1 canal configuration in Mx2Ps (approximately 75%), with 2 canals in 24% and 3 in only 1% of cases. However, studies from South Asia reveal significant inter-population discrepancies in canal morphology [5].

Various techniques have been employed to investigate root canal anatomy, including tooth sectioning, conventional radiographs, direct observation under magnification, tooth clearing, and computed tomography. CBCT has emerged as a valuable tool for the in vivo examination of root canal morphology, offering detailed, three-dimensional information on the internal anatomy of teeth [6].

In developing countries like Pakistan, however, the widespread use of CBCT is limited by factors such as cost, availability, and concerns regarding radiation exposure. As a result, conventional radiographic techniques like the Same Lingual Opposite Buccal (SLOB) rule are more commonly employed, albeit with significantly lower diagnostic accuracy compared to CBCT [7].

Therefore, the present study aimed to evaluate the variations in root canal morphology of maxillary second premolars and their association with gender using CBCT in a Pakistani population, recognizing that most existing morphological data are based on Western populations, which may not be representative due to ethnic and anatomical diversity.

## Materials and methods

Study settings

This retrospective cross-sectional study was conducted in the Department of Operative Dentistry and Endodontics at Bakhtawar Amin Dental Hospital, Multan. Ethical approval was obtained from the College of Physicians and Surgeons Pakistan, Research Evaluation Unit (CPSP/REU/DSG-2021-294-3653) prior to data collection.

Sample size and sampling technique

The sample size was calculated using OpenEpi software (www.OpenEpi.com), based on the findings of Celikten et al. [8], who reported a 49% prevalence of Vertucci Type I root canal morphology in Mx2P. With a 95% confidence level and a 5% margin of error, the minimum required sample size was determined to be 384. A consecutive non-probability sampling technique was employed to select the scans.

Inclusion and exclusion criteria

High-quality oral and maxillofacial CBCT scans of patients aged 18 to 65 years with fully developed roots of Mx2P were included. Scans of teeth that were restored, endodontically treated, grossly carious, fractured, ankylosed, or with visible pulpal calcifications were excluded.

CBCT analysis

A total of 384 Mx2Ps were selected from the CBCT records, comprising scans from 117 males and 105 females. All CBCT images were acquired using a standardized protocol and evaluated using Carestream Dental Imaging Software 3D module v2.4 (Carestream Health, Inc., Rochester, NY, USA).

**Figure 1:**
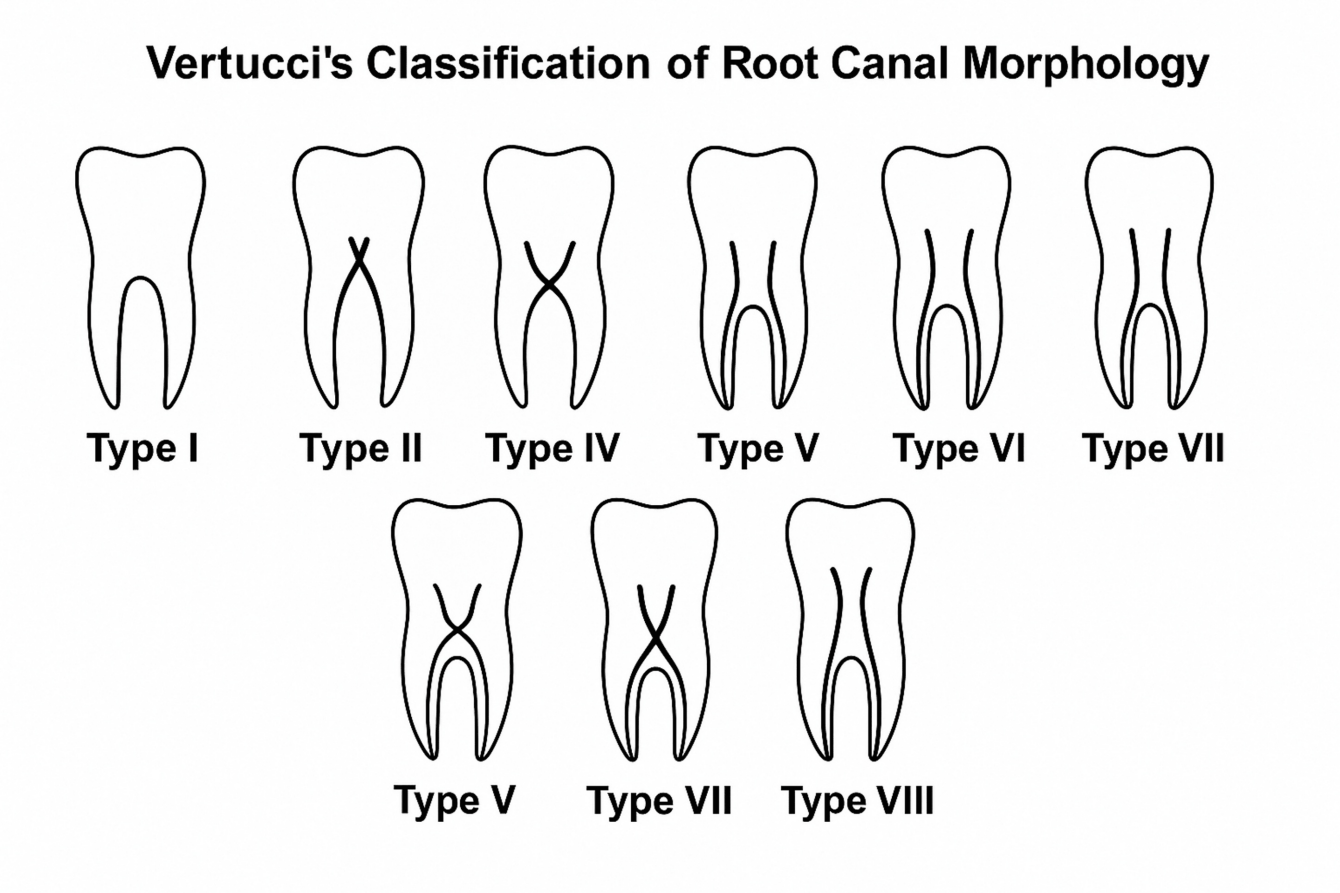
Vertucci’s classification of root canal morphology Type I: A single canal extends from the pulp chamber to the apex. Type II: Two separate canals leave the pulp chamber and merge to form one canal at the apex. Type III: One canal leaves the pulp chamber, divides into two within the root, and then merges to exit as one canal. Type IV: Two separate and distinct canals extend from the pulp chamber to the apex. Type V: One canal leaves the pulp chamber and divides into two separate canals with separate apical foramina. Type VI: Two canals leave the pulp chamber, merge in the middle, and then redivide into two separate canals before the apex. Type VII: One canal leaves the pulp chamber, divides and rejoins, and finally redivides into two canals near the apex. Type VIII: Three separate and distinct canals extend from the pulp chamber to the apex. *The image was created by the authors of this study.

The root canal morphology of each tooth was assessed and classified according to Vertucci’s classification system [7], which describes eight distinct types of canal configurations (Figure 1):

All assessments were performed by a single calibrated investigator (the principal author). To assess intra-examiner reliability, 30 randomly selected Mx2P scans were re-evaluated two weeks after the initial assessment. Intra-observer agreement was analyzed using Cohen’s Kappa statistic, which indicated excellent reproducibility (κ > 0.80).

Data analysis

All data were entered and analyzed using IBM SPSS Statistics for Windows, Version 25.0 (IBM Corp., Armonk, NY, USA). Descriptive statistics (means and standard deviations) were calculated for quantitative variables such as age. Frequencies and percentages were computed for categorical variables, including gender and root canal morphology types. The chi-square test was used to evaluate associations between gender and root canal morphology. A p-value < 0.05 was considered statistically significant.

## Results

The mean age of the subjects included in the study was 42.3 ± 13.7 years. A total of 384 maxillary second premolars (Mx2P) were evaluated, of which 205 (53.4%) were from male patients and 179 (46.6%) from female patients.

**Figure 2:**
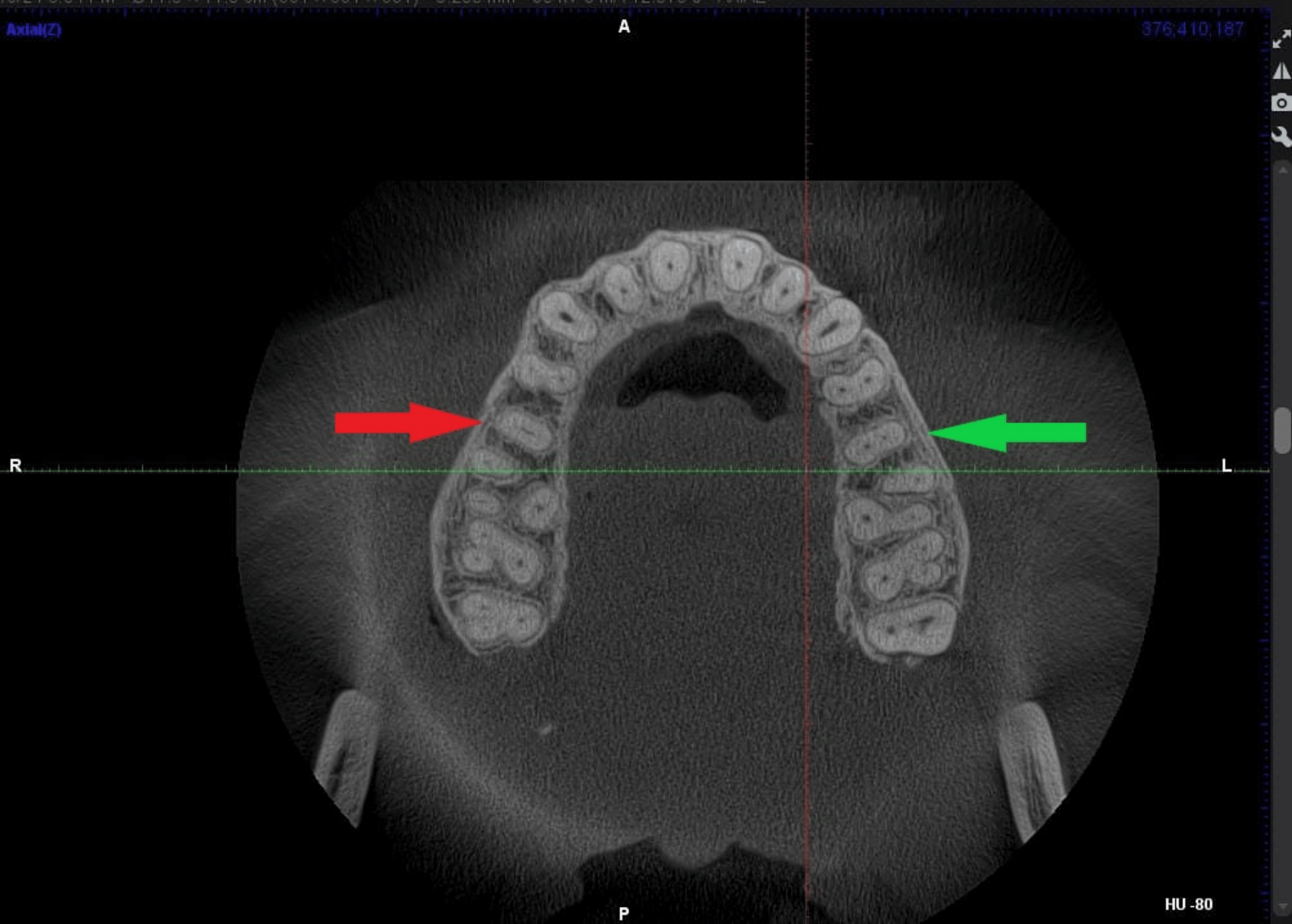
CBCT axial view showing Vertucci's Type I root canal morphology in the right upper maxillary second premolar (red arrow) and Vertucci's Type II root canal morphology in the left upper maxillary second premolar (green arrow) CBCT: cone-beam computed tomography

The most frequently observed root canal configuration was Vertucci’s Type I (Figure 2), identified in 240 teeth (62.5%). This was followed by Type II (Figure 2) in 96 teeth (25.0%) and Type IV in 30 teeth (7.8%), making them the three most prevalent morphologies.

**Figure 3:**
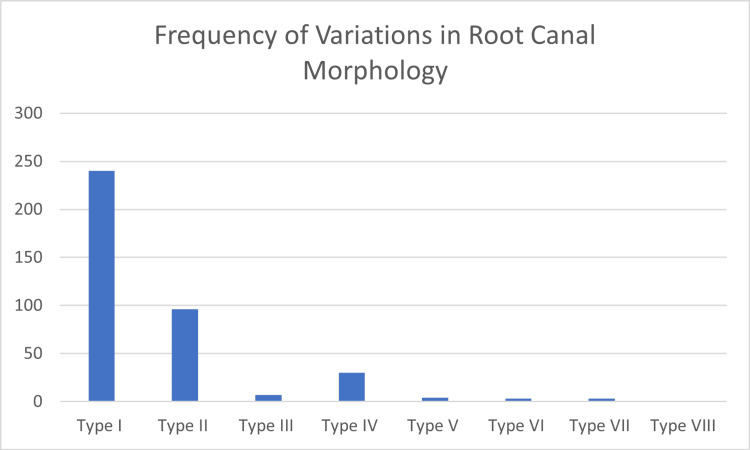
Frequency of variations in root canal morphology of maxillary second premolars according to Vertucci’s classification

Less common types included Type III in 7 teeth (1.8%), Type V in 4 teeth (1.0%), Type VI and Type VII in 3 teeth each (0.8%), and Type VIII in 1 tooth (0.3%) (Figure 3).

Statistical analysis using the chi-square test revealed no significant association between root canal morphology and gender (p = 0.915), as shown in Table 1.

**Table 1:** Distribution of root canal morphological types according to gender (n = 384) Chi-square test applied; significance level set at p < 0.05

Gender	Type I	Type II	Type III	Type IV	Type V	Type VI	Type VII	Type VIII	Total	Chi-square p-value
Male	131	49	3	17	2	1	1	1	205	0.915
Female	109	47	4	13	2	2	2	0	179	
Total	240	96	7	30	4	3	3	1	384	

## Discussion

Successful endodontic treatment requires a comprehensive understanding of root canal anatomy and morphology. All root canals must be accurately located, accessed, thoroughly debrided, disinfected, shaped, and obturated to ensure a favorable prognosis [9]. Treatment failures are often attributed to undetected canals, inadequate cleaning, or insufficient obturation [8]. Therefore, meticulous clinical and radiographic examination is essential for endodontic success [9].

The present study utilized CBCT to evaluate the root canal morphology of Mx2P in a Pakistani population, based on Vertucci’s classification system. CBCT has emerged as a superior, non-destructive imaging modality for in vivo three-dimensional visualization of root canal systems. Compared to traditional techniques, such as sectioning, clearing, or resin casting, CBCT offers higher accuracy and reproducibility without damaging the samples [10].

Our findings revealed that Vertucci's Type I canal configuration was the most prevalent, observed in 62.5% of the evaluated Mx2P, followed by Type II (25%) and Type IV (7.8%). These results are consistent with previous studies conducted in Middle Eastern and South Asian populations. For instance, Al Zubaidi et al. reported Type I in 60.4% of Saudi patients, while Asheghi et al. found Type I in 69.2% and Type II in 21% of an Iranian cohort [11,12]. Vertucci himself, in his original study, documented Type I in 48%, Type II in 22%, and Type IV in 11% of maxillary second premolars [13].

In European populations, studies have reported different trends: Caliskan et al. found Type I in 44% of Turkish patients [14]. Jayasimha Raj et al. in India reported lower prevalence of Type I (29.2%) and higher percentages of Type II (33.6%) and Type IV (31.1%) [15]. Abella et al. found only 39.3% Type I in Spanish patients [16]. Interestingly, our findings differ from studies in some populations. Bulut et al. observed only 14.3% Type I and a relatively high percentage of Type IV (25%) in a German cohort, suggesting increased canal complexity in European groups compared to Middle Eastern or South Asian populations [17]. In our study, no statistically significant association was found between root canal morphology and gender (p = 0.915), consistent with findings from earlier investigations. This reinforces the notion that gender may not play a significant role in determining root canal configuration. Similarly, Weng et al. found Type II (36%) and Type IV (33.8%) as the most frequent types in a Chinese population [18]. These discrepancies emphasize the ethnic and geographical variation in root canal morphology, reinforcing the need for population-specific data to guide clinical decisions.

Despite its strengths, the current study has several limitations. The study focused exclusively on maxillary second premolars, limiting the generalizability of findings to other teeth. Although the sample was drawn from a Pakistani population, ethnic diversity within the country may not be fully represented. The study found no association between gender and canal configuration, a limitation consistent with many previous investigations. Future research should include larger, community-based samples, explore other tooth types, and evaluate correlations between crown morphology and root canal anatomy. Actually, the small sample size and single-centered approach were the main limitations of the study.

Clinicians should remain vigilant of anatomical complexities in Mx2P and consider advanced imaging techniques like CBCT, especially when conventional methods are inconclusive. A thorough understanding of root canal morphology can help reduce endodontic failures associated with missed canals and incomplete treatment. Future multicenter studies with larger, ethnically diverse samples and inclusion of other tooth types are recommended to further elucidate anatomical patterns and improve the predictability of endodontic outcomes in clinical practice.

## Conclusions

This study highlights the predominance of Vertucci Type I root canal configuration in Mx2P in a Pakistani population, followed by Type II and Type IV. The use of CBCT proved valuable in accurately identifying and categorizing root canal morphologies in three dimensions, reinforcing its role as a reliable diagnostic tool in endodontics.

No significant association was found between gender and root canal morphology, indicating that canal configuration is likely independent of sex in this population. When compared to global data, variations in the prevalence of different Vertucci types were observed, underlining the importance of population-specific anatomical knowledge in enhancing the success of root canal therapy.
